# Supplementary material for: The Feasibility and Impact of Practising Online Forest Bathing to Improve Anxiety, Rumination, Social Connection and Long-COVID Symptoms: A Pilot Study
Source: Int J Environ Res Public Health. 2022 Nov 12;19(22):14905. doi: 10.3390/ijerph192214905 (PMC9691063; doi:10.3390/ijerph192214905)
Supplement: Supplementary file 1 [file ijerph-19-14905-s001.zip › ijerph-1974527-supplementary.pdf]

Long Covid symptoms
To what extent are you currently experiencing the following long-Covid symptoms?

extreme tiredness (fatigue)
Not at all 1 2 3 4 5 6 7 Very much so

shortness of breath
Not at all 1 2 3 4 5 6 7 Very much so

chest pain or tightness
Not at all 1 2 3 4 5 6 7 Very much so

problems with memory and concentration ("brain fog")
Not at all 1 2 3 4 5 6 7 Very much so

difficulty sleeping (insomnia)
Not at all 1 2 3 4 5 6 7 Very much so

heart palpitations
Not at all 1 2 3 4 5 6 7 Very much so

dizziness
Not at all 1 2 3 4 5 6 7 Very much so

pins and needles
Not at all 1 2 3 4 5 6 7 Very much so

joint pain
Not at all 1 2 3 4 5 6 7 Very much so

depression and anxiety
Not at all 1 2 3 4 5 6 7 Very much so

tinnitus, earaches
Not at all 1 2 3 4 5 6 7 Very much so

feeling sick, diarrhoea, stomach aches, loss of appetite
Not at all 1 2 3 4 5 6 7 Very much so

a high temperature, cough, headaches, sore throat
Not at all 1 2 3 4 5 6 7 Very much so

changes to sense of smell or taste
Not at all 1 2 3 4 5 6 7 Very much so

rashes
Not at all 1 2 3 4 5 6 7 Very much so
